# Progression-Free Survival as Early Efficacy Endpoint in Resectable Esophageal Cancer Treated With Neoadjuvant Therapy: A Systematic Review

**DOI:** 10.3389/fonc.2021.771546

**Published:** 2022-01-17

**Authors:** Jie Zhu, Jin Tao, Zhen Dai, Yan Tan, Li Jiang, Qifeng Wang, Jinyi Lang

**Affiliations:** ^1^ Radiation Oncology Key Laboratory of Sichuan Province, Sichuan Cancer Hospital & Institute, Sichuan Cancer Center, School of Medicine, University of Electronic Science and Technology of China, Chengdu, China; ^2^ Department of Radiation Oncology, Sichuan Cancer Hospital & Institute, Sichuan Cancer Center, School of Medicine, University of Electronic Science and Technology of China, Chengdu, China; ^3^ Department of Research and Development, Chengdu Institute of Biological Products Co., Ltd, Chengdu, China; ^4^ Department of Human Immunodeficiency Virus and Sexually Transmitted Diseases Control & Prevention, Chengdu Center for Disease Control & Prevention, Chengdu, China

**Keywords:** esophageal cancer, neoadjuvant therapy, progression-free survival, early efficacy endpoint, surrogate endpoint

## Abstract

To investigate literature-based evidence regarding progression-free survival (PFS) as an early efficacy endpoint in patients with resectable esophageal or gastroesophageal junction (GEJ) cancer receiving neoadjuvant therapy, this study identified large-scale randomized controlled trials (RCTs) with strict quality control. Twenty-four RCTs involving 7,514 patients were included. Trial-level correlation analysis was conducted to analyze the relationship between PFS hazard ratio (HR) and overall survival (OS) HR, Δ median PFS and Δ median OS. Correlation analysis at the neoadjuvant treatment arm level was performed between 1- to 5-year PFS and 5-year OS, median PFS and median OS. Subgroup analysis was performed in patients treated with standard neoadjuvant chemoradiotherapy (NCRT). The correlation was evaluated using the Pearson correlation coefficient *r* in weighted linear regression, with weight equal to patient size. In trial-level correlation, PFS were strongly associated with OS HR (*r*, 0.82 [95% confidence interval (CI), 0.42-0.97]) and Δ median survival (*r*, 0.83 [95% CI, 0.54-0.96]). In neoadjuvant treatment arms, there was a strong correlation between 1 to 5-year PFS rates and 5-year OS (*r*, 0.83-0.95), and median PFS and median OS (*r*, 0.97 [95% CI, 0.85-0.99]). NCRT subgroup analysis demonstrated acceptable consistency. In conclusion, we recommend PFS as an early efficacy endpoint in resected esophageal or GEJ cancer treated with neoadjuvant therapy.

## Introduction

Esophageal or gastroesophageal junction (GEJ) cancer is the seventh most common cancer worldwide, causing an estimated 509,000 deaths in 2018 (1). Multimodal treatment consisting of neoadjuvant chemoradiotherapy (NCRT) and surgical resection has been the standard treatment for resectable esophageal or GEJ cancer in recent years. However, even after standard NCRT plus surgical resection, about one-third of patients experience distant metastasis with or without local recurrence, which has poor outcomes with post-progression survival ranging from months to a few years (2). The real-world survival in patients with esophageal or GEJ cancer is far from satisfactory, with a 5-year survival rate of 47% for localized stage and 25% for regionally advanced stage (3). There is an urgent need to find more effective neoadjuvant therapies to improve the long-term survival in patients with resectable esophageal or GEJ cancer.

Overall survival (OS) has been considered the gold standard endpoint in randomized controlled trials (RCTs). However, an extended follow-up period and a large sample size are required to observe significant survival benefits when using OS as the primary endpoint, leading to high costs and long delays in introducing novel drugs. Effective post-progression treatment can reduce or even eliminate the apparent benefit of local tumor control and long-term recurrence, which results in non-significant OS prolongation. The evaluation of early efficacy endpoints, such as progression-free survival (PFS) or disease-free survival (DFS), requires a smaller sample size and shorter evaluation time than OS, which allows the implementation of more RCTs and accelerates the approval of novel drugs. Postoperative nivolumab maintenance has shown significant survival benefits in resectable esophageal or GEJ cancer, and the addition of immune checkpoint inhibitors (ICIs) to NCRT has been widely investigated. If the early efficacy endpoint of PFS is successfully established in resectable esophageal or GEJ cancer, the exploration of preoperative use of ICIs will be significantly accelerated.

However, previous studies demonstrated a poor trial-level correlation between PFS/DFS and OS in resectable esophageal or GEJ cancer treated with neoadjuvant therapy, indicating PFS/DFS as an unsuitable early efficacy endpoint (4, 5). However, these studies may not be comprehensive because they did not exclude small-scale RCTs or perform quality control before statistical analysis. Unqualified RCTs may confound a true correlation between the early efficacy endpoint and OS. Therefore, in this study, we only included large-scale RCTs and performed strict quality control for potentially eligible RCTs before correlation analysis. The primary aim of this study was to investigate PFS as an early efficacy endpoint in patients with resectable esophageal or GEJ cancer receiving neoadjuvant therapy through literature-based analysis at both trial and treatment arm levels. The secondary aim was to explore the association between pathological complete response (pCR), R0, and OS in patients treated with neoadjuvant therapy.

## Methods

### Literature Search and Quality Control

#### Inclusion and Exclusion Criteria

This study was exempted from review by the institutional review board because it used published data, and no human subjects were enrolled. The eligibility criteria included RCTs investigating long-term survival in patients with resectable esophageal or GEJ cancer who underwent neoadjuvant therapy followed by surgical resection. Studies were excluded if they met any of the following criteria: inoperable patients, inadequate survival data, sample size < 100 participants, non-epithelial histology (e.g., sarcomas or lymphomas), and non-English publications.

#### Literature Search

Studies published between 1 January 1990 and 31 December 2020 were identified through a systematic literature search of PubMed, Embase, the Cochrane Library, and Web of Science using the following search terms: (“esophageal” or “esophagus” or “esophagogastric” or “gastroesophageal”) AND (“cancer” or “carcinoma”) AND (“neoadjuvant” or “preoperative”) while restricting to RCT in article type. A manual search of each RTCs’ reference lists was also performed to include other potentially eligible RCTs. An independent literature search was performed by J.Z. and J.T., and further reviewed by the third author Q.F.W. Disagreements regarding study inclusion were resolved by J.Z., J.T., and Q.F.W.

#### Quality Control

According to the Cochrane Collaboration tool, the quality of potentially eligible RCTs weas assessed in the following seven domains: random sequence generation, allocation concealment, blinding of participants and personnel, blinding of outcome assessment, incomplete outcome data, selective reporting, and other biases. All available information from formal publications, meeting abstracts, and trial registries at ClinicalTrials.gov (www.clinicaltrials.gov) were integrated to draw a conclusion of low, unclear, or high risk of bias in each domain. RCTs with a high risk of bias in any domain were excluded from the statistical analysis.

Surgery is the major treatment for esophageal or GEJ carcinoma. A low surgery rate may indicate low compliance of participants, severe toxicity of neoadjuvant therapy, flaw in trial design, or immature surgical skill. Patients who first received neoadjuvant therapy and then failed or refused to undergo surgery were also included in the intention-to-treat (ITT) population. In this study, all survival outcomes were based on the ITT population. Therefore, a low surgery rate could not truly reflect the real prognosis of neoadjuvant treatment. We defined surgery resection rate as the proportion of patients that underwent surgery resection in the ITT population. RCTs with surgical resection rates < 80% were ranked with a high risk of bias in the domain of other biases.

### Statistical Methods

#### Endpoint Definition

OS was defined as the time interval from randomization to death from any cause. PFS was generally measured from the time of randomization or study entry to progression, recurrence, or death. DFS was defined heterogeneously among trials. A total of 10 RCTs reported DFS with a clear definition, among which 5 defined DFS as the time from a landmark of 6 months after randomization to incomplete resection, recurrence, or death, while the other 5 calculated DFS from randomization or surgery (**Supplemental Table 1**). Relapse-free survival (RFS) was calculated from randomization to the first event of local recurrence, distant recurrence, or death from any cause. Considering the homogenous definition of PFS, we investigated the potential eligibility of PFS as an early efficacy endpoint to replace OS in this study. For trials that only reported DFS or RFS, DFS or RFS was regarded as PFS approximately in the statistical analysis.

#### Data Extraction

Patient characteristics, sample size, primary endpoint, median follow-up time, standard and treatment arms, pCR rate, R0 resection rate, PFS hazard ratio (HR), OS HR, median PFS, median OS, PFS rates at different time points (1-, 2-, 3-, and 5-year), and 5-year OS were extracted. For a repeatedly reported RCT, we only included the latest results with the longest follow-up time. All survival outcomes were based on ITT population. If survival outcomes were not reported in the full text directly, HRs or survival rates at the different year points were extracted from the Kaplan–Meier survival curves (labeled “*”) using Engauge Digitizer software, according to methods detailed by Tierney et al. (6)

#### Correlation Evaluation

Correlation analyses of the RCTs were performed at both the trial and neoadjuvant treatment arm levels. At the trial level, survival benefit was represented by HR and Δ median survival time. Δ median survival time was defined as the absolute difference in the median survival of the treatment arm minus the median survival of the standard arm (Δ median survival = median survival of treatment arm – median survival of standard arm). The trial-level correlation relationship was evaluated using Pearson correlation coefficient *r* in weighted linear regression, with weight that depended on trial sample size.

At the neoadjuvant treatment arm level, only arms with neoadjuvant therapy were included in the analysis. The linear correlations between (1) median PFS and median OS; (2) PFS rates at different year points (1-, 2-, 3-, and 5-year) and 5-year OS; (3) pCR rate and median OS; (4) pCR rate and 5-year OS; (5) R0 resection rate and median OS; (6) R0 resection rate and 5-year OS were also evaluated by the correlation coefficient *r*, with weight equal to the sample size of each arm.

A strong linear correlation was indicated when *r* ≥ 0.8 (7). The 95% confidence interval (CI) of *r* was obtained using the bootstrap method with 1,000 replications. Statistical analysis was performed using SPSS statistical software (version 21.0, Armonk, NY: IBM Corp.), and data visualization was performed using the ggplot2 package in R software (version 3.3.2, R Foundation for Statistical Computing). For original data, please contact littlecancer@163.com.

#### Subgroup Analysis

To assess the consistency of correlation relationship in different patient populations. Subgroup analysis of the correlation between PFS and OS was performed as followings: (1) neoadjuvant strategy, NCRT vs. neoadjuvant chemotherapy (NCT); (2) pathological type, squamous cell carcinoma (SCC) vs. adenocarcinoma (AC); (3) publication year, 1996-2010 vs. 2011-2019. Correlation relationships were also evaluated using Pearson correlation coefficient *r* of weighted linear regression.

## Results

### RCTs Inclusion and Quality Assessment

**Figure 1:**
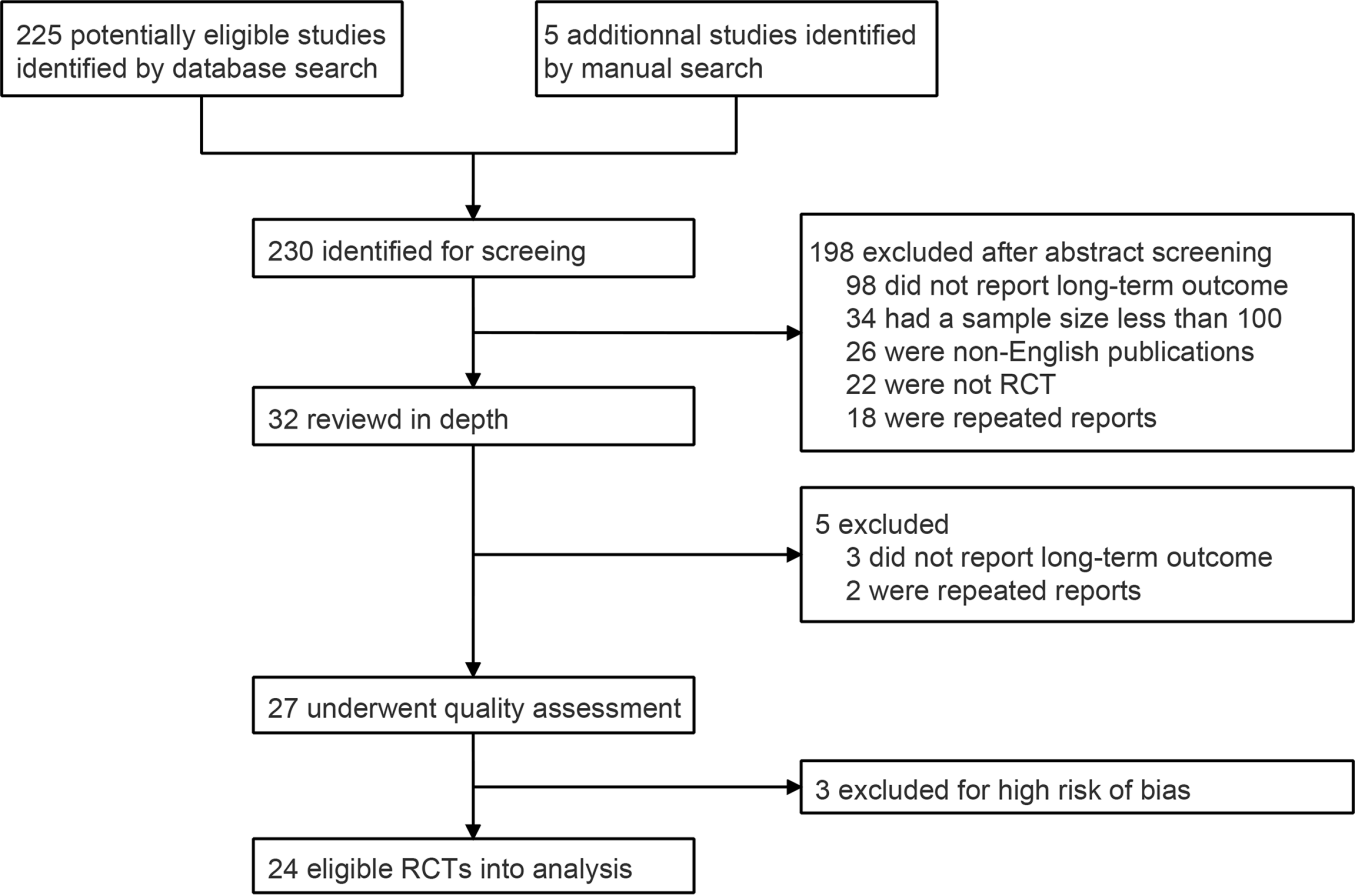
PRISMA flow chart for RCTs inclusion. RCT, randomized controlled trial.

A total of 230 abstracts were identified through database and manual searches. After excluding 198 ineligible records, the full texts of 32 records were reviewed in depth. Five unqualified records were excluded after full-text review, and 27 RCTs were included in the quality assessment (**Figure 1**). Seventeen trials were rated with unclear risk of selection bias because the randomization sequence generation and/or allocation concealment processes were not comprehensively reported. Three RCTs were excluded, because they had a low surgical resection rate (< 80%), which could introduce a high risk of bias of long-term survival based on the ITT population (**Supplemental Figure 1** and **Supplemental Table 2**) (8–10). Finally, 24 qualified RCTs were included in the statistical analysis (**Table 1**) (11–34).

**Table 1:** Summary of randomized controlled trials included in trial and treatment arm level analyses.

Trial	Inclusion criteria	Primary endpoint	Median FU (month)	No.	Treatment	pCR (%)	R0 (%)	PFS						OS		
								HR	Median (month)	1-y (%)	2-y (%)	3-y (%)	5-y (%)	HR	Median (month)	5-y (%)
NCRT + surgery vs. surgery alone (n = 8)																
NEOCRTEC5010 (Yang, 2018) (11)	SCC; age 18-70; T1-4N1M0 and T4N0M0; KPS ≥ 90	OS	40.8	224	NCRT + surgery	43.2*	98.4*	0.58*^P^ (DFS)	100.1*	85.1	77.8	70.9	63.1	0.71*^P^	100.1*	60.8
			34.8	227	Surgery	NA	91.2*		41.8*	75.1	60	52.1	45.8		66.5*	51.1
CROSS (Shapiro, 2015) (12)	SCC and AC; age ≤ 75; T1N1M0 and T2-3N0-1M0; PS ≤ 2	OS	84	178	NCRT + surgery	NA	92*	0.64*^P^	37.7*	71*	60*	51*	44*	0.68*^P^	48.6*	47*
				188	Surgery	NA	69*		16.2*	54*	41*	35*	27*		24*	33*
FFCD 9901 (Mariette, 2014) (13)	SCC and AC; age ≤ 75; T1-2N0-1M0 and T3N0M0; PS 0-1	OS	93.6	98	NCRT + surgery	33.3*	93.8*	0.92*^N^ (DFS)	27.8*	71.2	51.8	40.8	35.6*	0.99*^N^	31.8*	41.1*
				97	Surgery	NA	92.1*		27.8*	75.3	58.7	44.7	27.7*		41.2*	33.8*
Lv, 2010 (14)	SCC; Age ≥ 40; stage II-III	PFS	45.6	80	NCRT + surgery	NA	97.4*	NA	46.5*	89.3*	75.0	61.3*	37.5	NA	53.0*	43.5*
				78	Surgery + CRT	NA	78.2*	NA	45*	89.1*	74.9	61.1*	37.2*	NA	48*	42.3*
				80	Surgery	NA	80*		32.5*	84.5*	60.6	49.3*	25.9*		36*	33.8*
Burmeister, 2005 (15)	SCC and AC; T1-3N0-1M0; PS ≤ 1	PFS	64.8	128	NCRT + surgery	16	80	0.82*^N^	16.0*	55.7	39.1	32.5	30.7	0.89*^N^	22.2*	26.6
				128	Surgery	NA	59		12*	50.1	33.8	26.3	23.2		19.3*	23.4
Urba, 2001 (16)	SCC, AC and adenosquamous carcinoma; age ≤ 75; resectable; KPS ≥ 60	NA	98.4	50	NCRT + surgery	28*	97.8	NA	9.84 (DFS)	47.1	32.7	28*	25.4	0.73*^N^	16.9*	20.3
				50	Surgery	NA	97.8		9.7	46.9	22.7	16*	11.6		17.6*	10.1
Bosset, 1997 (17)	SCC; age ≤ 70; T1-3N0M0 and T1-2N1M0; PS ≤ 2	OS	55.2	143	NCRT + surgery	26*	81.2	0.6*^P^ (DFS)	19.7	63.7	44.5	40	31.4	1*^N^	18.6*	25.2
				139	Surgery	NA	68.6		8.4	44.9	32.2	27.3	24.7		18.6*	24.1
Walsh, 1996 (18)	AC; age ≤ 76; TanyNanyM0; PS ≤ 2	OS	9.6	58	NCRT + surgery	25*	NA	NA	NA	NA	NA	NA	NA	NA	16.0*	NA
			8.4	55	Surgery	NA	NA		NA	NA	NA	NA	NA		11.0*	NA
NCT + surgery vs. surgery alone (n = 5)																
Boonstra, 2011 (19)	SCC; T1-3NanyM0; M1a (distal); age < 80; KPS > 70	OS	15.6	85	NCT + surgery	NA	71*	0.72*^P^ (DFS)	5.4	46.5	38.8	32.5	28.1	0.71*^P^	16.0*	26*
			14.4	84	Surgery	NA	57*		NA	29.5	19.1	17.8	13.4		12*	17*
Ychou, 2011 (20)	AC; age 18-75; PS ≤ 2	OS	68.4	113	NCT + surgery	NA	87*	0.65*^P^ (DFS)	20.0	67.9	46.8	40.0	34*	0.69*^P^	31.6	38*
				111	Surgery	NA	74*		12.2	49.2	30.8	25.1	19*		22.3	24*
OEO2 (Allum, 2009) (21)	SCC, AC and undifferentiated; resectable	OS	70.8	400	NCT + surgery	NA	60*	0.82*^P^ (DFS)	7.0	42.0	29.6	24.1	19.8	0.84*^P^	17.8	23*
			73.2	402	Surgery	NA	54*		NA	31.8	21.9	17.4	14.4		14.5	17.1*
RTOG 8911 (Kelsen, 2007) (22)	SCC and AC; age ≥ 18; T1-3NanyM0	OS	105.6	213	NCT + surgery	2.5*	62*	0.95^N^ (DFS)	NA	38.9	22	16.6	14.4	1.07*^N^	14.9*	20.6
				227	Surgery	NA	59*		NA	28.9	20.4	16.5	14.3		16.1*	21.8
Law, 1997 (23)	SCC; TanyNanyM0	OS	16.8	74	NCT + surgery	6.7*	NA	NA	8.0*	NA	NA	NA	NA	NA	16.8*	NA
				73	Surgery	NA	NA		9.7*	NA	NA	NA	NA		13.0*	NA
NCT vs. postoperative CT (n = 2)																
JCOG9907 (Ando, 2012) (24)	SCC; Age ≤ 75; stage II-III (excluding T4); PS ≤ 2	PFS	61.2	164	NCT + surgery	2.4*	94*	0.84*^N^	35.8	70.7	57.5	49.5	44*	0.73*^P^	NA	55*
				166	Surgery + CT	NA	91*		23.5	66.6	48.3	42.1	39*		36.8	43*
NCT01225523 (Zhao, 2015) (25)	SCC; age ≥ 18; resectable; PS ≤ 1	RFS	60	175	NCT + surgery + CT	NA	NA	0.62*^P^ (RFS)	23.0*	75.0	47.4	40.5	35*	0.79*^P^	29.04*	38*
				171	NCT + surgery	NA	NA		15*	64.3	32.0	24.5	19.1*		22.0*	22*
Induction CT + NCRT/NCT vs. NCRT/NCT (n =2)																
NCT00525915 (Ajani, 2013) (26)	SCC and AC; T1N+M0 and T2–3NanyM0; age ≤ 75; PS ≤ 1	pCR	NA	54	Induction CT + NCRT + surgery	26*	NA	NA	NA	NA	NA	NA	NA	NA	43.68*	48.4
				55	NCRT + Surgery	13*	NA		NA	NA	NA	NA	NA		45.6*	45.4
POET (Stahl, 2017) (27)	AC; T3-4; PS ≤ 1	OS	126	60	Induction CT + NCRT + surgery	14.3*	88*	0.64*^N^	NA	NA	NA	NA	NA	0.65*^N^	30.8*	39.5*
				59	NCT + Surgery	1.9*	NA		NA	NA	NA	NA	NA		21.1*	24.4*
NCRT vs. NCT (n =1)																
NCT01362127 (von Döbeln, 2019) (28)	SCC and AC; T1N1 or T2-3N0-1 and M0-1a; age ≤ 75; PS ≤ 1	pCR	NA	90	NCRT + surgery	28	87	1.02*^N^	24	65	50.6	44*	38.9*	1.09*^N^	31.4*	42.2*
				91	NCT + surgery	9	74		20.0	64.8	48.7	44*	33*		36*	39.6*
Different NCT regimens (n =2)																
OE05 (Alderson, 2017) (29)	AC; T1-2N1M0 and T3-4N0-1M0; age ≤ 75; PS ≤ 1	OS	76.8	446	ECX + surgery	7*	66*	0.84*^N^	21.4*	NA	NA	NA	NA	0.9*^N^	26.2*	31.7
				451	CF + surgery	1*	59*		18.4*	NA	NA	NA	NA		23.4*	28.9
OGSG1003 (Yamasaki, 2017) (30)	SCC; T1-4aNanyM0-1LYM(supraclavicular); age ≥ 20; PS ≤ 1	RFS	34.8	81	DCF + surgery	NA	96.2*	0.53*^P^ (RFS)	NA	73.0	64.1*	62.1	NA	0.62^N^	NA	NA
			34.8	81	ACF + surgery	NA	95.9*		10.8	47.1	42.9*	41.5	NA		NA	NA
Conventional chemotherapy + targeted drugs (n =2)																
SAKK 75/08 (Ruhstaller, 2018) (31)	SCC and AC; T2N1-3M0 and T3-4aNanyM0; age 18-75; PS ≤ 1	PFS	48	149	Cetuximab + induction CT + NCRT + surgery	37*	95*	0.79*^N^	34.8*	74*	58*	50*	46.4	0.73*^N^	61.2*	52.4
				151	Induction CT + NCRT + surgery	33*	97*		24*	73*	50*	41*	35.5		36*	41.5
NCT00450203 (Cunningham, 2017) (32)	AC; resectable; age ≥ 18; PS ≤ 1	OS	39.6	530	Bevacizumab + perioperative CT + surgery	3*	61*	1.05*^N^	NA	NA	NA	NA	NA	1.09*^N^	33.6	35.6
			36	533	Perioperative CT + surgery	5*	64*		NA	NA	NA	NA	NA		33.5	40.2
Minimally invasive surgery vs. open surgery (n = 2)																
ROBOT (van der Sluis, 2019) (33)	SCC and AC; T1-4aN0-3M0; age 18-80; PS ≤ 2	Postoperative complication	39.6	54	NCRT/NCT + RAMIE surgery	NA	93*	0.99^N^ (DFS)	28.0*	65.5	54.0	48.7	48.7	1.05^N^	NA	49.1
				55	NCRT/NCT + open surgery	NA	96*		28.0*	68.5	51.1	48.4	NA		NA	NA
TIME (Straatman, 2017) (34)	SCC and AC; T1-3N0-1M0; age 18-75; PS ≤ 2	DFS	27.6	59	NCRT/NCT + MIE	NA	91.5*	0.69*^N^ (DFS)	17.0	70.4	43.6	40.2*	31.9	0.88*^N^	27	37.5
			21.6	56	NCRT/NCT + open surgery	NA	83.9*		16.9	54.7	40.3	35.9*	25.4		21.8	30

The standard arm is labeled in bold. *Represents data directly reported in the full text. “P” and “N” in the top right of the HR indicate positive and negative result, respectively. AC, adenocarcinoma; ACF, cisplatin, fluorouracil and adriamycin; CF, cisplatin and fluorouracil; CT, chemotherapy; DCF, cisplatin, fluorouracil and docetaxel; DFS, disease-free survival; ECX, epirubicin, cisplatin and capecitabine; FU, follow-up; HR, hazard ratio; KPS, Karnofsky performance score; MIE, minimally invasive esophagectomy; NA, not available; NCRT, neoadjuvant chemoradiotherapy; NCT, neoadjuvant chemotherapy; OS, overall survival; pCR, pathologic complete response; PFS, progression-free survival; PS, performance score; RAMIE, robot-assisted minimally invasive thoracolaparoscopic esophagectomy; RFS, relapse-free survival; SCC, squamous cell carcinoma.

### Characteristics of Included RCTs

A total of 24 RCTs with 7,514 patients were included in the analysis. The median sample size was 231 participants, and the median follow-up time ranged from 8.4 to 126 months. The most common primary endpoint was OS (n = 13, 54%), followed by PFS (n = 4, 17%), RFS (n = 2, 8%), pCR (n = 2, 8%), DFS (n = 1, 4%), and postoperative complications (n = 1, 4%) (**Table 1**). The majority of RCTs (n = 16, 67%) were followed up every 3-4 months during the first 1-2 years (**Supplemental Table 1**).

According to the purpose of the study, RCTs were classified into eight subgroups: (1) eight RCTs (33%) compared NCRT plus surgery with surgery alone; (2) five (21%) compared NCT plus surgery with surgery alone; (3) two (8%) compared NCT with postoperative chemotherapy; (4) two (8%) focused on induction chemotherapy; (5) one (4%) compared NCRT with NCT; (6) two (8%) investigated different NCT regimens; (7) two (8%) compared targeted therapy with conventional chemotherapy; (8) two (8%) focused on surgical methods, comparing minimally invasive surgery with open surgery (**Table 1**).

### Trial-Level Correlation of Treatment Benefit Between PFS and OS

**Figure 2:**
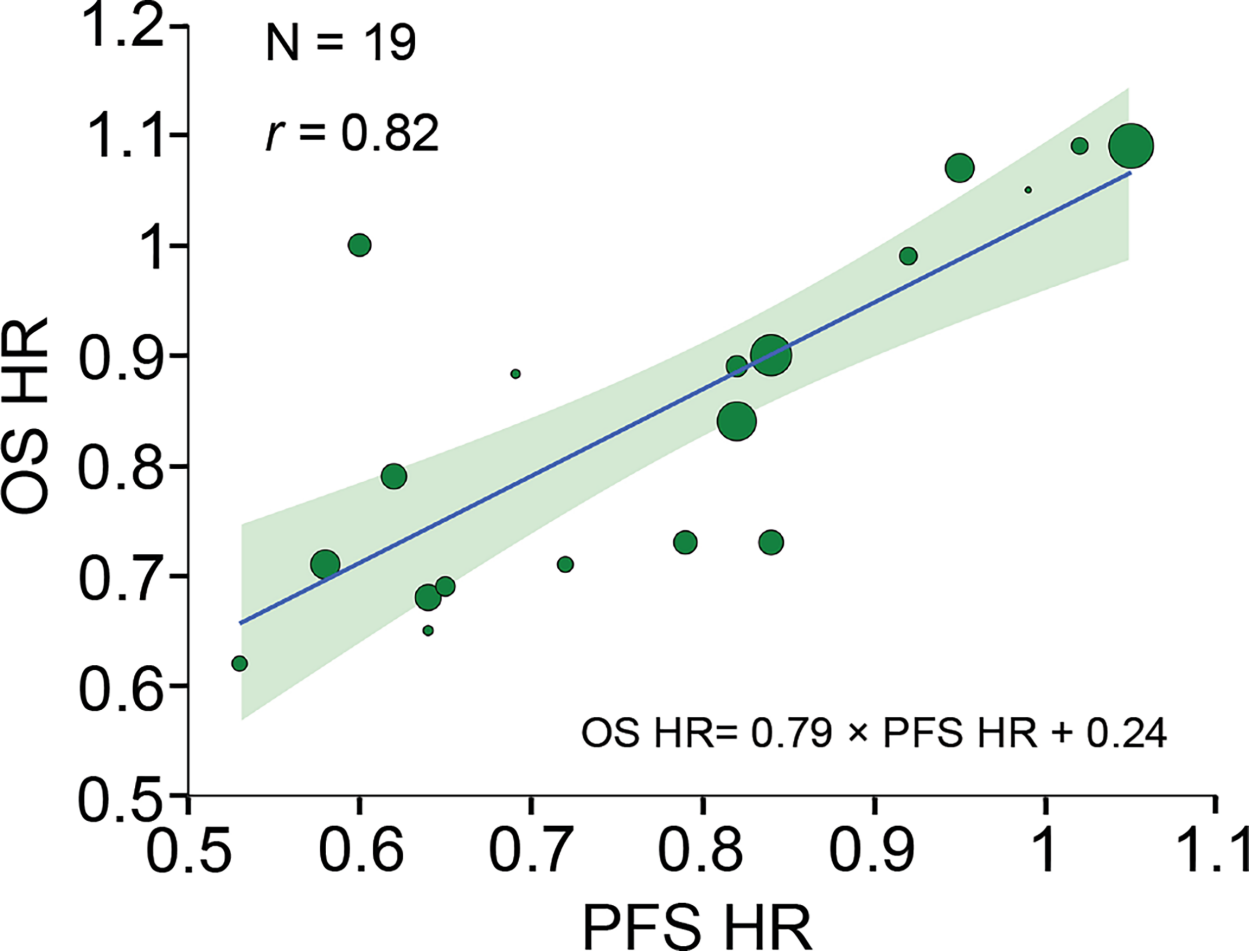
Trial-level correlation between PFS HR and OS HR. Circle size is proportional to the number of patients included in each comparison. The solid blue line indicates the fitted weighted linear regression line; the light green zone represents its 95% CI; *r* indicates the correlation coefficient. PFS, progression-free survival; OS, overall survival; HR, hazard ratio; CI, confidence interval.

**Figure 3:**
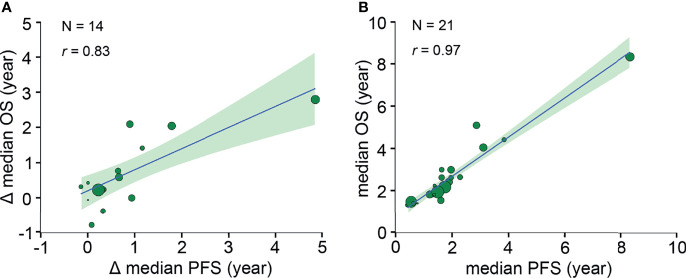
Trial- and neoadjuvant treatment arm-level correlations between median PFS and median OS. **(A)** Trial-level correlation between Δ median PFS and Δ median OS. Δ median survival time is defined as the absolute difference between the median survival of treatment arm and the median survival of standard arm. **(B)** Neoadjuvant treatment arm-level correlation between median PFS and median OS. Circle size is proportional to the number of patients in each arm. The solid blue line indicates the fitted weighted linear regression line; the light green zone represents its 95% CI; *r* indicates the correlation coefficient. PFS, progression-free survival; OS, overall survival; CI, confidence interval.

A strong correlation was found after analyzing 19 pairs of PFS HR and OS HR (*r*, 0.82 [95% CI, 0.42-0.97]) (**Figure 2**). Fourteen pairs of Δ median PFS and Δ median OS were reported, and Δ median PFS was strongly correlated with Δ median OS (*r*, 0.83 [95% CI, 0.54-0.96]) (**Figure 3A**).

### Neoadjuvant Treatment Arm-Level Correlation Between PFS and OS

Twenty-one pairs of median PFS and median OS were reported, and median PFS was strongly correlated with median OS (*r*, 0.97 [95% CI, 0.85-0.99]) (**Figure 3B**).

**Figure 4:**
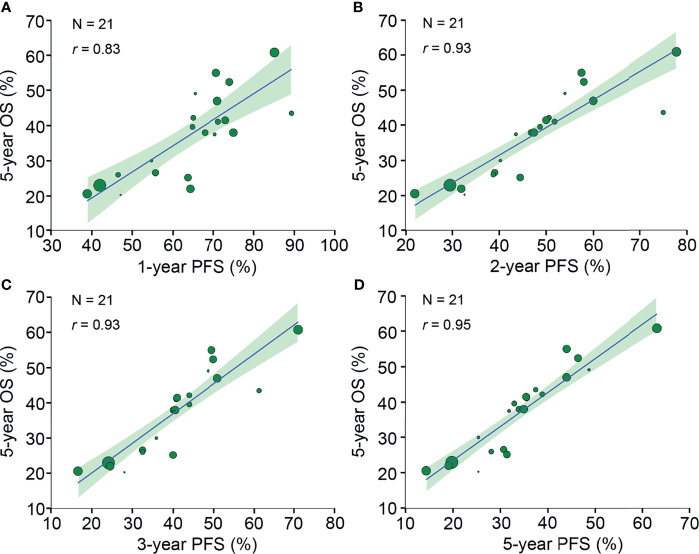
Neoadjuvant treatment arm-level correlation between PFS rates and 5-year OS. The neoadjuvant treatment arm-level association between **(A)** 1-year PFS and 5-year OS, **(B)** 2-year PFS and 5-year OS, **(C)** 3-year PFS and 5-year OS, and **(D)** 5-year PFS and 5-year OS. Circle size is proportional to the number of patients in each treatment arm. The solid blue line indicates the fitted weighted linear regression line; the light green zone represents its 95% CI; r indicates the correlation coefficient. PFS, progression-free survival; OS, overall survival; CI, confidence interval.

Twenty-nine neoadjuvant treatment arms reported 5-year OS, of which 21 (72%) treatment arms reported PFS rates at 1-5 years. The 1-year PFS (*r*, 0.83 [95% CI, 0.63-0.94]), 2-year PFS (*r*, 0.93 [95% CI, 0.81-0.98]), 3-year PFS (*r*, 0.93 [95% CI, 0.82-0.98]) and 5-year PFS (*r*, 0.95 [95% CI, 0.89-0.98]) were all strongly correlated with 5-year OS (**Figure 4**).

### Subgroup Analysis of the Correlation Between PFS and OS

In subgroup analysis of neoadjuvant strategy, 14 and 10 trials investigated NCRT and NCT, respectively. Both NCRT and NCT subgroups showed similar and consistent trial- and arm-level correlation relationships with overall trials (**Supplemental Table 3**).

For pathological subgroup, 8, 5, and 11 RCTs enrolled SCC, AC, and SCC or AC patients, respectively. Due to a limited number of trial- and arm-level data available in SCC and AC subgroups, correlation coefficient *r* and its CI varied greatly and lacked reliability (**Supplemental Table 3**).

Eight and 16 RCTs were published in 1996-2010 and 2011-2019, respectively. In 1996-2010 subgroup, only 4 pairs of HRs were reported and a poor trial-level correlation was concluded. In 2011-2019 subgroups, trial- and arm-level correlations were consistent with overall trials (**Supplemental Table 3**).

### Neoadjuvant Treatment Arm-Level Correlation Among pCR, R0, and OS

Twenty-nine neoadjuvant treatment arms from 24 RCTs reported median OS, of which 20 (69%) and 22 (76%) arms reported pCR and R0 resection rates, respectively. The pCR rate (*r*, 0.66 [95% CI, -0.04-0.87]) (**Supplemental Figure 2A**) and R0 resection rate (*r*, 0.60 [95% CI, 0.20-0.78]) (**Supplemental Figure 2B**) did not demonstrate a strong correlation with median OS.

Twenty-nine neoadjuvant treatment arms reported 5-year OS, of which 19 (66%) and 24 (83%) treatment arms reported pCR and R0 resection rates, respectively. The pCR rate (*r*, 0.54 [95% CI, -0.12-0.85]) (**Supplemental Figure 2C**) and R0 resection rate (*r*, 0.68 [95% CI, 0.38-0.86]) (**Supplemental Figure 2D**) did not show a strong linear correlation with 5-year OS.

## Discussion

This large-scale, comprehensive study included high-quality RCTs to investigate the association between early efficacy endpoints and OS in patients with resectable esophageal or GEJ cancer who underwent neoadjuvant therapy followed by surgical resection. In contrast with previous findings, analyses of 24 qualified RCTs in this study demonstrated that PFS was strongly correlated with OS at both trial and neoadjuvant treatment arm levels. PFS benefits can be translated into OS prolongation. The PFS rate at 1-5 years and median PFS were highly predictive of the 5-year OS and median OS, respectively. The correlation relationships of the NCRT and NCT subgroups were generally consistent with overall trials. For patients with resectable esophageal or GEJ cancer receiving neoadjuvant therapy, our findings provide new evidence supporting the clinical use of PFS as an early efficacy endpoint to evaluate survival benefits and accelerate approval for superior treatment regimens. These findings may improve patient prognosis and advance the field by allowing novel drugs to enter the market more rapidly.

In clinical practice, NCRT is a state-of-the-art treatment modality for resectable esophageal or GEJ cancer. There was a very low pCR rate (< 10%) in patients receiving NCT alone. The addition of preoperative radiotherapy to chemotherapy significantly reduced tumor size and improved the pCR and R0 resection rates. Once NCRT patients had achieved pCR, a remarkably lower risk of recurrence, especially in the regional lymph node and lung, could be expected, and NCRT patients with R1 resection had a notably higher recurrence rate than those with R0 resection (35). The pCR and R0 resection were directly indicated for a lower risk of recurrence. For patients undergoing NCRT followed by surgery, 71% of recurrences occurred within the first 2 years of surgery and the median time to the first recurrence was only 11 months (35). NCRT significantly decreased early locoregional and distant progressions, and the risk reduction in early progression has been successfully translated into significant survival prolongation (12). Because of the poor effectiveness of conventional chemotherapy, the majority of patients with advanced esophageal cancer cannot survive for more than 1 year (36). For resectable esophageal or GEJ cancer patients undergoing NCRT/NCT plus surgery, the high risk of early recurrence and poor post-progression survival corresponded well with the strong linear correlation between PFS and OS in this study; the improvement in PFS would be confidently converted into survival prolongation, suggesting an effective clinical application of PFS as an early efficacy endpoint in the conventional chemotherapy era.

Recently, anti-programmed death-1 (PD-1) antibody therapy has significantly prolonged survival in both advanced and resectable esophageal or EGJ cancer patients (36–39). Compared with conventional NCRT, the risk of early recurrence was significantly reduced by adding nivolumab postoperatively, with the median DFS of almost 2 years (39). Post-progression survival was also significantly improved by applying anti-PD-1 antibody therapy (36–38). This predictive model was mainly based on the result of conventional chemoradiotherapy; therefore, efforts to extrapolate to the efficacy of immune therapy should be preceded with caution. The correlation relationship should be modified and optimized in the modern era of immunochemotherapy.

Previous literature-based studies indicated that early efficacy endpoints of PFS or DFS were poorly associated with OS at the trial level. The treatment benefit of PFS or DFS was not likely to be converted to survival benefit in resectable esophageal or GEJ cancer patients receiving neoadjuvant therapy (4, 5). In this study, the most recent trials were updated and only large-scale RCTs with ≥ 100 patients were included. The surgical resection rate was also included in the quality assessment, and only trials with a resection rate ≥80% were eligible for final inclusion. RCTs were later assessed with comprehensive quality control, and 3 RCTs with high risk of bias were excluded from final analysis. Through strict inclusion criteria and quality assessment, original data was guaranteed with high quality and a low risk of bias, and the correlation relationships concluded in this study were believed to be reliable.

There were some limitations to this study. First, this was a literature-based systematic review without the possibility to assess individual patient data. In subgroup analysis of pathological type, 11 trials enrolled both SCC and AC. The main long-term survival of patients with different pathological types was reported as an integral. These patients could not be classified into SCC or AC subgroup, leading to the lack of data in pathological type subgroup analysis. therefore, an individual-level subgroup analysis was encouraged in further study. Second, a standardized definition of survival endpoints and surveillance strategies was required for precise modeling, which was not feasible in this literature-based study. DFS was measured heterogeneously, from randomization or landmarks at 6 months after randomization. Although we primarily investigated PFS as an early efficacy endpoint in this study, DFS was regarded as the approximate value of PFS in nine RCTs without PFS data. Moreover, the exact date of disease progression or relapse is difficult to determine clinically and always lies in the interval between two consecutive imaging assessments. In this study, the patients in the majority of RCTs were followed up every 3 months within the first 2 years with computerized tomography and/or endoscopy (**Supplemental Table 1**), but the inherent heterogeneity in the follow-up frequency and imaging assessment was still present and could not be removed.

In conclusion, for patients with resectable esophageal or GEJ cancer receiving neoadjuvant therapy, our assessment of a large sample of high-quality data provides high-level evidence that PFS is a valid early efficacy endpoint for OS. Our finding may accelerate the development of neoadjuvant therapy in resectable esophageal or EGJ cancer by early approvement of superior treatment regimens and rapid market introduction of novel drugs.

## Data Availability Statement

The original contributions presented in the study are included in the article/**Supplementary Material**. Further inquiries can be directed to the corresponding author.

## Author Contributions

QW designed the study and revised the manuscript. JZ performed literature search, collected raw data, performed statistical analysis, and drafted the manuscript. JT performed literature search. ZD supervised statistical analysis. YT and JL supervised study design. LJ collected raw data. All authors contributed to the article and approved the submitted version.

## Funding

This work was supported by the Science and Technology Department of Sichuan Province (grant nos. 2019YFS0378, 2018JY0277, and 2020YJ0453), CSCO-Genecast Oncology Research Fund (Grant No. Y-2019Genecast-041), and the Cancer Research Foundation of China Anti-cancer Association for Young Scientists (Grant No. CAYC18A33).

## Conflict of Interest

Author JT was employed by Chengdu Institute of Biological Products Co., Ltd.

The remaining authors declare that the research was conducted in the absence of any commercial or financial relationships that could be construed as a potential conflict of interest.

## Publisher’s Note

All claims expressed in this article are solely those of the authors and do not necessarily represent those of their affiliated organizations, or those of the publisher, the editors and the reviewers. Any product that may be evaluated in this article, or claim that may be made by its manufacturer, is not guaranteed or endorsed by the publisher.

## Supplementary Material

Supplementary Figure 1 Summary of risk of bias in RCTs. “+” (green), “?” (yellow), and “−” (red) represent low, unclear, and high risk of bias, respectively. RCT, randomized controlled trial.

Supplementary Figure 2 Neoadjuvant treatment arm-level correlation among pCR rate, R0 resection rate and OS. The neoadjuvant treatment arm-level association between **(A)** pCR rate and median OS, **(B)** R0 resection rate and median OS, **(C)** pCR rate and 5-year OS, and **(D)** R0 resection rate and 5-year OS. Circle size was proportional to the number of patients in each treatment arm. The solid blue line indicates the fitted weighted linear regression line, the light green zone represents its 95% CI, and *r* indicates the correlation coefficient. pCR, pathological complete response; OS, overall survival; CI, confidence interval.
