# Supplementary material for: Progression-Free Survival as Early Efficacy Endpoint in Resectable Esophageal Cancer Treated With Neoadjuvant Therapy: A Systematic Review
Source: Front Oncol. 2022 Jan 17;11:771546. doi: 10.3389/fonc.2021.771546 (PMC8801608; doi:10.3389/fonc.2021.771546)
Supplement: Supplementary Figure 1 — Summary of risk of bias in RCTs. “+” (green), “?” (yellow), and “−” (red) represent low, unclear, and high risk of bias, respectively. RCT, randomized controlled trial. [file DataSheet_1.docx]

| **PICOS** | |
| --- | --- |
| **Population** | Resectable esophageal or GEJ cancer patients |
| **Intervention** | Neoadjuvant therapy plus surgery |
| **Comparators** | Not applicable |
| **Outcomes** | The correlation between early endpoint and OS |
| **Study designs** | Systematic review of RCTs |
