# Supplementary material for: Progression-Free Survival as Early Efficacy Endpoint in Resectable Esophageal Cancer Treated With Neoadjuvant Therapy: A Systematic Review
Source: Front Oncol. 2022 Jan 17;11:771546. doi: 10.3389/fonc.2021.771546 (PMC8801608; doi:10.3389/fonc.2021.771546)
Supplement: Supplementary file 3 [file DataSheet_3.docx]

**Supplemental Table 1. The definition of disease-free survival, progression-free survival and follow-up strategy in randomized controlled trials.**

| Trial | DFS | | | PFS | | | Follow-up | |
| --- | --- | --- | --- | --- | --- | --- | --- | --- |
|  | Starting point | Event | | Starting point | | Event | Frequency | Method |
| ***NCRT+ surgery vs. surgery alone (n = 8)*** | | | | | | | | |
| NEOCRTEC5010 (Yang, 2018)^1^ | R0 resection | Disease recurrence or death | | | NA | NA | Every 3 months in the first year, every 6 months thereafter | CT and clinical examination |
| CROSS (Shapiro, 2015)^2^ | NA | NA | | | Randomization | Primary irresectability, locoregional recurrence, distant dissemination, or death | Every 3 months in the first year, every 6 months in the second year, and annually thereafter until 5 years | NA |
| FFCD 9901 (Mariette, 2014)^3^ | Randomization | Locoregional recurrence, metastatic or death | | | NA | NA | Every 4 months in the first 2 years, every 6 months for the next 2 years, then annually | CT and clinical examination |
| Lv, 2010^4^ | NA | NA | | | Study entry | Progression or death | Every 3 months in the first 2 years, every 6 months thereafter | endoscopy, endoscopic ultrasonography and CT |
| Burmeister, 2005^5^ | NA | NA | | | Randomization | If CR or free of disease after surgery: relapse or death; If not macroscopically free of disease after surgery: at the time of surgery or at the time the decision was made not to proceed to surgery. | Every 3 months in the first 2 years, every 6 months thereafter | Investigations to detect relapse were not done routinely, unless patients had signs of recurrence. |
| Urba, 2001^6^ | NA | NA | | | NA | NA | NA | NA |
| Bosset, 1997^7^ | NA | NA | | | NA | NA | Every 4 months after surgery until death or the end of the study period. | Clinical examination, esophagography, chest radiography, and ultrasonography of the liver |
| Walsh, 1996^8^ | NA | NA | | | NA | NA | NA | NA |
| ***NCT + surgery vs. surgery alone (n = 5)*** | | | | | | | | |
| Boonstra, 2011^9^ | 6 months after randomization | | Macroscopically incomplete resection, local and distant recurrence, and death | NA | | NA | Every 3 to 4 months in the first year, every 6 months in the second year, and annually thereafter until 5 years | Recurrence of disease was diagnosed on clinical grounds. When a relapse was suspected, radiologic, endoscopic, or histologic confirmation was performed |
| Ychou, 2011^10^ | 6 months after randomization | | Incomplete resection, local and distant recurrence, and death | NA | | NA | Every 6 months for 5 years | Clinical examination, tumor marker CA 19-9, and abdominal ultrasound or CT scan |
| OEO2 (Allum, 2009)^11^ | 6 months after randomization | | Macroscopically incomplete resection, local and distant recurrence, and death | NA | | NA | Every 3 months in the first year, every 6 months thereafter | NA |
| RTOG 8911 (Kelsen, 2007)^12^ | Randomization | | Local, regional, or distant failure or death; patients without an R0 or R1 resection were considered as local failures at day 1 | NA | | NA | NA | NA |
| Law, 1997^13^ | NA | | NA | NA | | NA | Monthly in the first year, every 3 months thereafter | Physical examination. Endoscopic, radiologic, and histopathologic examinations were performed if recurrence was suspected |
| ***NCT vs. postoperative CT (n = 2)*** | | | | | | | | |
| JCOG9907 (Ando, 2012)^14^ | NA | NA | | Randomization | | Relapse or death | NA | NA |
| NCT01225523 (Zhao, 2015)^15^ | NA | NA | | NA | | NA | Every 3 months in first 2 years and every 6 months thereafter | Whenever a relapse was suspected, radiologic, endoscopic, or histologic confirmation was compulsory |
| ***Induction CT + NCRT/NCT vs. NCRT/NCT (n =2)*** | | | | | | | | |
| NCT00525915 (Ajani, 2013)^16^ | NA | NA | | NA | | NA | Every 3 months for 1 year, every 6 months for two additional years, and annually for up to 5 years | Imaging studies and endoscopic evaluations (every other visit) |
| POET (Stahl, 2017)^17^ | NA | NA | | Randomization | | Disease progression or death | Every 3 months within 2 years, every 6 months thereafter until 5 years | Patients’ history, clinical examination, blood counts and chemistry, ECG, thoracic X-ray, abdominal ultrasound and upper intestinoscopy, CT |
| ***NCRT vs. NCT (n =1)*** | | | | | | | | |
| NCT01362127 (von Döbeln, 2019)^18^ | NA | NA | | Registration | | Progression or death | Every 3 months during  the first 2 years, then every 6 months until 5 years | CT and/or endoscopy |
| ***Different NCT regimens (n =2)*** | | | | | | | | |
| OE05 (Alderson, 2017)^19^ | 1 week after the last patient had surgery, up to a maximum of 6 months from assignment | Local recurrence, distant metastases, or death | | Randomization | | Local or distant recurrence, or death | Every 3 months in the first year, every 6 months in the second year, then annually | NA |
| OGSG1003 (Yamasaki, 2017) ^20^ | NA | RFS: locoregional or distant recurrence | | NA | | NA | Every 3 months during the first 2 years, every 6 months for the next 3 years, and annually after 5 years | NA |
| ***Conventional chemotherapy + targeted drugs (n =2)*** | | | | | | | | |
| SAKK 75/08 (Ruhstaller, 2018)^21^ | NA | NA | | Randomization | | Progression, recurrence, or death | Every 3 months following surgery or after the end of neoadjuvant treatment of the first 3 years and then every 6 months until 5 years | Clinical and radiological examinations |
| NCT00450203 (Cunningham, 2017)^22^ | 6 months after randomization | R2 resection, no resection, recurrence or death | | Randomization | | Recurrence or death | Every 6 months within the first 3 years and annually thereafter | NA |
| ***Minimally invasive surgery vs. open surgery (n = 2)*** | | | | | | | | |
| ROBOT (van der Sluis, 2019)^23^ | Surgery | Recurrence or death related to disease and/or treatment or last date of follow-up | | NA | | NA | NA | NA |
| TIME (Straatman, 2017)^24^ | Surgery | Recurrence or death | | NA | | NA | Every 6 months within the first 5 years | NA |

Abbreviation: CR, complete response; CT, chemotherapy; DFS, disease-free survival; ECG, electrocardiograph; NA, not available; NCRT, neoadjuvant chemoradiotherapy; NCT, neoadjuvant chemotherapy; PFS, progression-free survival.

**Supplemental Table 2.** **The assessment of risk of bias in randomized controlled trials by using the Cochrane Collaboration tool.**

| Trial | Bias | Risk of bias | Support for judgement |
| --- | --- | --- | --- |
| ***NCRT + surgery vs. surgery alone (n = 9)*** | | | |
| NEOCRTEC5010 (Yang, 2018)^1^ | Random sequence generation (selection bias) | Low risk | Random assignment was generated by computer generated random assignment lists at the Sun Yat-sen University Cancer Center Clinical Trial Center. |
|  | Allocation concealment (selection bias) | Low risk | The assignments were placed in sealed envelopes, labeled by stratum, which would only be unsealed after patient registration. |
|  | Blinding of participants and personnel (performance bias) | Low risk | No blinding, but the survival outcome was not likely to be influenced by lack of blinding. |
|  | Blinding of outcome assessment (detection bias) | Low risk | No blinding, but the survival outcome was not likely to be influenced by lack of blinding. |
|  | Incomplete outcome data (attrition bias) | Low risk | Censoring was unlikely to introduce bias for survival outcome. |
|  | Selective reporting (reporting bias) | Low risk | All prespecified endpoint outcomes were reported. |
|  | Other bias | Low risk | 185/224 (82.6%) participants in the arm NCRT + surgery and 227/227 (100%) participants in the arm surgery received resection. The study appeared to be free of other sources of bias. |
| CROSS (Shapiro, 2015)^2^ | Random sequence generation (selection bias) | Low risk | Randomisation was done centrally at the Clinical Trial Center at Erasmus MC (Rotterdam, the Netherlands), by computer-generated randomisation lists for each stratum, with random permuted block sizes of four or six. |
|  | Allocation concealment (selection bias) | Low risk | Central randomization, participants and investigators can’t foresee assignment. |
|  | Blinding of participants and personnel (performance bias) | Low risk | No blinding, but the survival outcome was not likely to be influenced by lack of blinding. |
|  | Blinding of outcome assessment (detection bias) | Low risk | No blinding, but the survival outcome was not likely to be influenced by lack of blinding. |
|  | Incomplete outcome data (attrition bias) | Low risk | Censoring was unlikely to introduce bias for survival outcome. |
|  | Selective reporting (reporting bias) | Low risk | All prespecified endpoint outcomes were reported. |
|  | Other bias | Low risk | 161/178 (90%) participants in the arm NCRT + surgery and 162/188 (86%) participants in the arm surgery received resection. The study appeared to be free of other sources of bias. |
| FFCD 9901 (Mariette, 2014)^3^ | Random sequence generation (selection bias) | Low risk | Randomization was performed centrally with a minimization technique that ensured equal distribution of patients regarding stratification factors. |
|  | Allocation concealment (selection bias) | Low risk | Central randomization, participants and investigators can’t foresee assignment. |
|  | Blinding of participants and personnel (performance bias) | Low risk | No blinding, but the survival outcome was not likely to be influenced by lack of blinding. |
|  | Blinding of outcome assessment (detection bias) | Low risk | No blinding, but the survival outcome was not likely to be influenced by lack of blinding. |
|  | Incomplete outcome data (attrition bias) | Low risk | Censoring was unlikely to introduce bias for survival outcome. |
|  | Selective reporting (reporting bias) | Low risk | All prespecified endpoint outcomes were reported. |
|  | Other bias | Low risk | 84/98 (85.7%) participants in the arm NCRT + surgery and 91/97 (93.8%) participants in the arm surgery received resection. The study appeared to be free of other sources of bias. |
| Lv, 2010^4^ | Random sequence generation (selection bias) | Low risk | The randomization method was based on random numerals produced by computer. |
|  | Allocation concealment (selection bias) | Unclear risk | Insufficient information available to permit a judgement of low risk or high risk. Because the method of concealment is not described. |
|  | Blinding of participants and personnel (performance bias) | Low risk | No blinding, but the survival outcome was not likely to be influenced by lack of blinding. |
|  | Blinding of outcome assessment (detection bias) | Low risk | No blinding, but the survival outcome was not likely to be influenced by lack of blinding. |
|  | Incomplete outcome data (attrition bias) | Low risk | 10 cases were lost. Censoring was unlikely to introduce bias for survival outcome. |
|  | Selective reporting (reporting bias) | Low risk | All prespecified endpoint outcomes were reported. |
|  | Other bias | Low risk | 76/80 (97.4%) participants in the arm NCRT + surgery and 64/80 (80%) participants in the arm surgery received radical resection. The study appeared to be free of other sources of bias. |
| Burmeister, 2005^5^ | Random sequence generation (selection bias) | Low risk | The random sequence was generated by use of minimisation by the trial statistician, and blocks of four were used. |
|  | Allocation concealment (selection bias) | Low risk | Patients were randomized by central telephone randomisation done by the trial coordinator at the NHMRC Clinical Trials Centre, Sydney, Australia. The allocation sequence was concealed to all central staffZz . |
|  | Blinding of participants and personnel (performance bias) | Low risk | No blinding, but the survival outcome was not likely to be influenced by lack of blinding. |
|  | Blinding of outcome assessment (detection bias) | Low risk | No blinding, but the survival outcome was not likely to be influenced by lack of blinding. |
|  | Incomplete outcome data (attrition bias) | Low risk | Censoring was unlikely to introduce bias for survival outcome. |
|  | Selective reporting (reporting bias) | Low risk | All prespecified endpoint outcomes were reported. |
|  | Other bias | Low risk | 105/128 (82%) participants in the arm NCRT + surgery and 110/128 (85.9%) participants in the arm surgery received resection. The study appeared to be free of other sources of bias. |
| Lee, 2004 (excluded)^25^ | Random sequence generation (selection bias) | Unclear risk | Insufficient information available to permit a judgement of low risk or high risk. Because the method of sequence generation is not described. |
|  | Allocation concealment (selection bias) | Unclear risk | Insufficient information available to permit a judgement of low risk or high risk. Because the method of concealment is not described. |
|  | Blinding of participants and personnel (performance bias) | Low risk | No blinding, but the survival outcome was not likely to be influenced by lack of blinding. |
|  | Blinding of outcome assessment (detection bias) | Low risk | No blinding, but the survival outcome was not likely to be influenced by lack of blinding. |
|  | Incomplete outcome data (attrition bias) | Low risk | Censoring was unlikely to introduce bias for survival outcome. |
|  | Selective reporting (reporting bias) | Low risk | All prespecified endpoint outcomes were reported. |
|  | Other bias | High risk | 35/51 (69%) participants in the arm NCRT + surgery and 48/50 (96%) participants in the arm surgery received resection. In ITT analysis, only 69% participants in the arm NCRT + surgery actually received resection, which has a high risk of bias in the outcome assessment. |
| Urba, 2001^6^ | Random sequence generation (selection bias) | Unclear risk | Insufficient information available to permit a judgement of low risk or high risk. Because the method of sequence generation is not described. |
|  | Allocation concealment (selection bias) | Unclear risk | Insufficient information available to permit a judgement of low risk or high risk. Because the method of concealment is not described. |
|  | Blinding of participants and personnel (performance bias) | Low risk | No blinding, but the survival outcome was not likely to be influenced by lack of blinding. |
|  | Blinding of outcome assessment (detection bias) | Low risk | No blinding, but the survival outcome was not likely to be influenced by lack of blinding. |
|  | Incomplete outcome data (attrition bias) | Low risk | Censoring was unlikely to introduce bias for survival outcome. |
|  | Selective reporting (reporting bias) | Low risk | All prespecified endpoint outcomes were reported. |
|  | Other bias | Low risk | 47/50 (94%) participants in the arm NCRT + surgery and 50/50 (100%) participants in the arm surgery received resection. The study appeared to be free of other sources of bias. |
| Bosset, 1997^7^ | Random sequence generation (selection bias) | Unclear risk | Insufficient information available to permit a judgement of low risk or high risk. Because the method of sequence generation is not described. |
|  | Allocation concealment (selection bias) | Low risk | Central randomization, participants and investigators can’t foresee assignment. |
|  | Blinding of participants and personnel (performance bias) | Low risk | No blinding, but the survival outcome was not likely to be influenced by lack of blinding. |
|  | Blinding of outcome assessment (detection bias) | Low risk | No blinding, but the survival outcome was not likely to be influenced by lack of blinding. |
|  | Incomplete outcome data (attrition bias) | Low risk | Censoring was unlikely to introduce bias for survival outcome. |
|  | Selective reporting (reporting bias) | Low risk | All prespecified endpoint outcomes were reported. |
|  | Other bias | Low risk | 138/143 (96.5%) participants in the arm NCRT + surgery and 137/139 (98.6%) participants in the arm surgery received resection. The study appeared to be free of other sources of bias. |
| Walsh, 1996^8^ | Random sequence generation (selection bias) | Unclear risk | Insufficient information available to permit a judgement of low risk or high risk. Because the method of sequence generation is not described. |
|  | Allocation concealment (selection bias) | Unclear risk | Insufficient information available to permit a judgement of low risk or high risk. Because the method of concealment is not described. |
|  | Blinding of participants and personnel (performance bias) | Low risk | No blinding, but the survival outcome was not likely to be influenced by lack of blinding. |
|  | Blinding of outcome assessment (detection bias) | Low risk | No blinding, but the survival outcome was not likely to be influenced by lack of blinding. |
|  | Incomplete outcome data (attrition bias) | Low risk | Censoring was unlikely to introduce bias for survival outcome. |
|  | Selective reporting (reporting bias) | Low risk | All prespecified endpoint outcomes were reported. |
|  | Other bias | Low risk | 52/58 (89.6%) participants in the arm NCRT + surgery and 55/55 (100%) participants in the arm surgery received resection. The study appeared to be free of other sources of bias. |
| ***NCT + surgery vs. surgery alone (n = 5)*** | | | |
| Boonstra, 2011^9^ | Random sequence generation (selection bias) | Unclear risk | Insufficient information about the sequence generation process available to permit a judgement of low risk or high risk. |
|  | Allocation concealment (selection bias) | Low risk | Central randomization, participants and investigators can’t foresee assignment. |
|  | Blinding of participants and personnel (performance bias) | Low risk | No blinding, but the survival outcome was not likely to be influenced by lack of blinding. |
|  | Blinding of outcome assessment (detection bias) | Low risk | No blinding, but the survival outcome was not likely to be influenced by lack of blinding. |
|  | Incomplete outcome data (attrition bias) | Low risk | Censoring was unlikely to introduce bias for survival outcome. |
|  | Selective reporting (reporting bias) | Low risk | All prespecified endpoint outcomes were reported. |
|  | Other bias | Low risk | 76/85 (89.4%) participants in the arm NCT + surgery and 82/84 (97.6%) participants in the arm surgery received resection. The study appeared to be free of other sources of bias. |
| Ychou, 2011^10^ | Random sequence generation (selection bias) | Low risk | Random assignment was performed by using minimization procedure. |
|  | Allocation concealment (selection bias) | Low risk | Eligible patients were randomly assigned to either preoperative chemotherapy followed by surgical resection (CS group) or surgical resection alone (S group) by phone call through the centralized randomization system of the Biostatistics and Epidemiology Department of the Gustave Roussy Institute. |
|  | Blinding of participants and personnel (performance bias) | Low risk | No blinding, but the survival outcome was not likely to be influenced by lack of blinding. |
|  | Blinding of outcome assessment (detection bias) | Low risk | No blinding, but the survival outcome was not likely to be influenced by lack of blinding. |
|  | Incomplete outcome data (attrition bias) | Low risk | Censoring was unlikely to introduce bias for survival outcome. |
|  | Selective reporting (reporting bias) | Low risk | All prespecified endpoint outcomes were reported. |
|  | Other bias | Low risk | 109/113 (96.4%) participants in the arm NCT + surgery and 110/111 (99%) participants in the arm surgery received resection. The study appeared to be free of other sources of bias. |
| OEO2 (Allum, 2009)^11^ | Random sequence generation (selection bias) | Unclear risk | Insufficient information available to permit a judgement of low risk or high risk. Because the method of sequence generation is not described. |
|  | Allocation concealment (selection bias) | Unclear risk | Insufficient information available to permit a judgement of low risk or high risk. Because the method of concealment is not described. |
|  | Blinding of participants and personnel (performance bias) | Low risk | No blinding, but the survival outcome was not likely to be influenced by lack of blinding. |
|  | Blinding of outcome assessment (detection bias) | Low risk | No blinding, but the survival outcome was not likely to be influenced by lack of blinding. |
|  | Incomplete outcome data (attrition bias) | Low risk | Censoring was unlikely to introduce bias for survival outcome. |
|  | Selective reporting (reporting bias) | Low risk | All prespecified endpoint outcomes were reported. |
|  | Other bias | Low risk | 357/400 (89%) participants in the arm NCT + surgery and 386/402 (96%) participants in the arm surgery received resection. The study appeared to be free of other sources of bias. |
| RTOG 8911 (Kelsen, 2007)^12^ | Random sequence generation (selection bias) | Unlcear risk | Insufficient information available to permit a judgement of low risk or high risk. Because the method of sequence generation is not described. |
|  | Allocation concealment (selection bias) | Unclear risk | Insufficient information available to permit a judgement of low risk or high risk. Because the method of concealment is not described. |
|  | Blinding of participants and personnel (performance bias) | Low risk | No blinding, but the survival outcome was not likely to be influenced by lack of blinding. |
|  | Blinding of outcome assessment (detection bias) | Low risk | No blinding, but the survival outcome was not likely to be influenced by lack of blinding. |
|  | Incomplete outcome data (attrition bias) | Low risk | Censoring was unlikely to introduce bias for survival outcome. |
|  | Selective reporting (reporting bias) | Low risk | All prespecified endpoint outcomes were reported. |
|  | Other bias | Low risk | 180/213 (84.5%) participants in the arm NCT + surgery and 218/227 (96%) participants in the arm surgery received resection. The study appeared to be free of other sources of bias. |
| Law, 1997^13^ | Random sequence generation (selection bias) | Unclear risk | Insufficient information available to permit a judgement of low risk or high risk. Because the method of sequence generation is not described. |
|  | Allocation concealment (selection bias) | Unclear risk | Insufficient information available to permit a judgement of low risk or high risk. Because the method of concealment is not described. |
|  | Blinding of participants and personnel (performance bias) | Low risk | No blinding, but the survival outcome was not likely to be influenced by lack of blinding. |
|  | Blinding of outcome assessment (detection bias) | Low risk | No blinding, but the survival outcome was not likely to be influenced by lack of blinding. |
|  | Incomplete outcome data (attrition bias) | Low risk | Censoring was unlikely to introduce bias for survival outcome. |
|  | Selective reporting (reporting bias) | Low risk | All prespecified endpoint outcomes were reported. |
|  | Other bias | Low risk | 66/74 (89%) participants in the arm NCT + surgery and 69/73 (95%) participants in the arm surgery received resection. The study appeared to be free of other sources of bias. |
| ***NCT vs. postoperative CT (n = 2)*** | | | |
| JCOG9907 (Ando, 2012)^14^ | Random sequence generation (selection bias) | Low risk | Minimization method. |
|  | Allocation concealment (selection bias) | Low risk | Central randomization, participants and investigators can’t foresee assignment. |
|  | Blinding of participants and personnel (performance bias) | Low risk | No blinding, but the survival outcome was not likely to be influenced by lack of blinding. |
|  | Blinding of outcome assessment (detection bias) | Low risk | No blinding, but the survival outcome was not likely to be influenced by lack of blinding. |
|  | Incomplete outcome data (attrition bias) | Low risk | Censoring was unlikely to introduce bias for survival outcome. |
|  | Selective reporting (reporting bias) | Low risk | All prespecified endpoint outcomes were reported. |
|  | Other bias | Low risk | 157/166 (94.5%) participants in the arm NCT + surgery and 149/164 (90.8%) participants in the arm Surgery + postoperative CT received resection. The study appeared to be free of other sources of bias. |
| NCT01225523 (Zhao, 2015)^15^ | Random sequence generation (selection bias) | Unclear risk | Insufficient information available to permit a judgement of low risk or high risk. Because the method of sequence generation is not described. |
|  | Allocation concealment (selection bias) | Unclear risk | Insufficient information available to permit a judgement of low risk or high risk. Because the method of concealment is not described. |
|  | Blinding of participants and personnel (performance bias) | Low risk | No blinding, but the survival outcome was not likely to be influenced by lack of blinding. |
|  | Blinding of outcome assessment (detection bias) | Low risk | No blinding, but the survival outcome was not likely to be influenced by lack of blinding. |
|  | Incomplete outcome data (attrition bias) | Low risk | Censoring was unlikely to introduce bias for survival outcome. |
|  | Selective reporting (reporting bias) | Low risk | All prespecified endpoint outcomes were reported. |
|  | Other bias | Low risk | 161/175 (92%) participants in the arm A and 159/172 (92.4%) participants in the arm B received resection. The study appeared to be free of other sources of bias. |
| ***Induction CT + NCRT/NCT vs. NCRT/NCT (n =2)*** | | | |
| NCT00525915 (Ajani, 2013)^16^ | Random sequence generation (selection bias) | Unclear risk | Insufficient information available to permit a judgement of low risk or high risk. Because the method of sequence generation is not described. |
|  | Allocation concealment (selection bias) | Low risk | The randomization was conducted using an in-house web-based software program that dynamically balanced the two groups for histology, baseline stage, gender, race, and age. |
|  | Blinding of participants and personnel (performance bias) | Low risk | No blinding, but the survival outcome was not likely to be influenced by lack of blinding. |
|  | Blinding of outcome assessment (detection bias) | Low risk | No blinding, but the survival outcome was not likely to be influenced by lack of blinding. |
|  | Incomplete outcome data (attrition bias) | Low risk | Censoring was unlikely to introduce bias for survival outcome. |
|  | Selective reporting (reporting bias) | Low risk | All prespecified endpoint outcomes were reported. |
|  | Other bias | Low risk | 55/63 (87%) participants in the arm A and 54/63 (85.7%) participants in the arm B received resection. The study appeared to be free of other sources of bias. |
| POET (Stahl, 2017)^17^ | Random sequence generation (selection bias) | Unclear risk | Insufficient information available to permit a judgement of low risk or high risk. Because the method of sequence generation is not described. |
|  | Allocation concealment (selection bias) | Low risk | Central randomization, participants and investigators can’t foresee assignment. |
|  | Blinding of participants and personnel (performance bias) | Low risk | No blinding, but the survival outcome was not likely to be influenced by lack of blinding. |
|  | Blinding of outcome assessment (detection bias) | Low risk | No blinding, but the survival outcome was not likely to be influenced by lack of blinding. |
|  | Incomplete outcome data (attrition bias) | Low risk | Censoring was unlikely to introduce bias for survival outcome. |
|  | Selective reporting (reporting bias) | Low risk | All prespecified endpoint outcomes were reported. |
|  | Other bias | Low risk | 52/59 (88%) participants in the arm A and 49/60 (81.6%) participants in the arm B received resection. The study appeared to be free of other sources of bias. |
| ***NCRT vs. NCT (n =1)*** | | | |
| NCT01362127 (von Döbeln, 2019)^18^ | Random sequence generation (selection bias) | Unclear risk | Patients were stratified by histological tumor type and randomized independently by a computerized software at the Regional Oncological Centre in Stockholm. Insufficient information available to permit a judgement of low risk or high risk. Because the method of sequence generation is not described. |
|  | Allocation concealment (selection bias) | Low risk | The allocation sequence was concealed to all investigators |
|  | Blinding of participants and personnel (performance bias) | Low risk | No blinding, but the survival outcome was not likely to be influenced by lack of blinding. |
|  | Blinding of outcome assessment (detection bias) | Low risk | No blinding, but the survival outcome was not likely to be influenced by lack of blinding. |
|  | Incomplete outcome data (attrition bias) | Low risk | Censoring was unlikely to introduce bias for survival outcome. |
|  | Selective reporting (reporting bias) | Low risk | All prespecified endpoint outcomes were reported. |
|  | Other bias | Low risk | Tumor resection rate was 87% in patients allocated to chemoradiotherapy and 86% in patients allocated to chemotherapy. The study appeared to be free of other sources of bias. |
| ***Different NCT regimens (n =3)*** | | | |
| OE05 (Alderson, 2017)^19^ | Random sequence generation (selection bias) | Low risk | Allocation was done using a computerized minimisation program, with a random element, and stratified by centre and tumour stage. |
|  | Allocation concealment (selection bias) | Low risk | Central randomization, participants and investigators can’t foresee assignment. |
|  | Blinding of participants and personnel (performance bias) | Low risk | No blinding, but the survival outcome was not likely to be influenced by lack of blinding. |
|  | Blinding of outcome assessment (detection bias) | Low risk | No blinding, but the survival outcome was not likely to be influenced by lack of blinding. |
|  | Incomplete outcome data (attrition bias) | Low risk | Censoring was unlikely to introduce bias for survival outcome. |
|  | Selective reporting (reporting bias) | Low risk | All prespecified endpoint outcomes were reported. |
|  | Other bias | Low risk | 411/451 (91%) participants in the arm CF and 387/446 (87%) participants in the arm ECX received resection. The study appeared to be free of other sources of bias. |
| OGSG1003 (Yamasaki, 2017)^20^ | Random sequence generation (selection bias) | Unclear risk | Insufficient information available to permit a judgement of low risk or high risk. Because the method of sequence generation is not described. |
|  | Allocation concealment (selection bias) | Low risk | Central randomization, participants and investigators can’t foresee assignment. |
|  | Blinding of participants and personnel (performance bias) | Low risk | No blinding, but the survival outcome was not likely to be influenced by lack of blinding. |
|  | Blinding of outcome assessment (detection bias) | Low risk | No blinding, but the survival outcome was not likely to be influenced by lack of blinding. |
|  | Incomplete outcome data (attrition bias) | Low risk | Censoring was unlikely to introduce bias for survival outcome. |
|  | Selective reporting (reporting bias) | Low risk | All prespecified endpoint outcomes were reported. |
|  | Other bias | Low risk | 74/81 (91%) participants in the arm ACF and 78/81 (96%) participants in the arm ACF received resection. The study appeared to be free of other sources of bias. |
| NCT00573131 (DeWitt, 2017) (excluded)^26^ | Random sequence generation (selection bias) | Unclear risk | Insufficient information available to permit a judgement of low risk or high risk. Because the method of sequence generation is not described. |
|  | Allocation concealment (selection bias) | Low risk | Central randomization, participants and investigators can’t foresee assignment. |
|  | Blinding of participants and personnel (performance bias) | Low risk | No blinding, but the survival outcome was not likely to be influenced by lack of blinding. |
|  | Blinding of outcome assessment (detection bias) | Low risk | No blinding, but the survival outcome was not likely to be influenced by lack of blinding. |
|  | Incomplete outcome data (attrition bias) | Low risk | Censoring was unlikely to introduce bias for survival outcome. |
|  | Selective reporting (reporting bias) | Low risk | All prespecified endpoint outcomes were reported. |
|  | Other bias | High risk | Only 51.4% and 56.9% participants in the treatment arm and standard arm respectively received resection. The low resection rate would lead to high risk of bias in the outcome assessment. |
| ***Conventional chemotherapy + targeted drugs (n =2)*** | | | |
| SAKK 75/08 (Ruhstaller, 2018)^21^ | Random sequence generation (selection bias) | Low risk | Randomization was centralized at the SAKK Coordinating Center with stratification by center, histological type, stage, and gender using the minimization method with 90% allocating probability |
|  | Allocation concealment (selection bias) | Low risk | Central randomization, participants and investigators can’t foresee assignment. |
|  | Blinding of participants and personnel (performance bias) | Low risk | No blinding, but the survival outcome was not likely to be influenced by lack of blinding. |
|  | Blinding of outcome assessment (detection bias) | Low risk | No blinding, but the survival outcome was not likely to be influenced by lack of blinding. |
|  | Incomplete outcome data (attrition bias) | Low risk | Censoring was unlikely to introduce bias for survival outcome. |
|  | Selective reporting (reporting bias) | Low risk | All prespecified endpoint outcomes were reported. |
|  | Other bias | Low risk | 132/149 (89%) participants in the arm Cetuximab and 130/151 (86%) participants in the arm Control received resection. The study appeared to be free of other sources of bias. |
| NCT00450203 (Cunningham, 2017)^22^ | Random sequence generation (selection bias) | Low risk | Sequence generation was performed by computer programme that implemented a minimization algorithm with a random element. |
|  | Allocation concealment (selection bias) | Low risk | Treatment allocation was done via a telephone call to the Medical Research Council Clinical Trials Unit at University College London. |
|  | Blinding of participants and personnel (performance bias) | Low risk | No blinding, but the survival outcome was not likely to be influenced by lack of blinding. |
|  | Blinding of outcome assessment (detection bias) | Low risk | No blinding, but the survival outcome was not likely to be influenced by lack of blinding. |
|  | Incomplete outcome data (attrition bias) | Low risk | Censoring was unlikely to introduce bias for survival outcome. |
|  | Selective reporting (reporting bias) | Low risk | All prespecified endpoint outcomes were reported. |
|  | Other bias | Low risk | 438/530 (83%) participants in the arm bevacizumab and 457/533 (86%) participants in the arm Control received resection. The study appeared to be free of other sources of bias. |
| ***Minimally invasive surgery vs. open surgery (n = 3)*** | | | |
| ROBOT (van der Sluis, 2019)^23^ | Random sequence generation (selection bias) | Low risk | Allocation of concealment was performed using computer generated random numbers in sealed opaque envelopes corresponding to either RAMIE or open surgery. |
|  | Allocation concealment (selection bias) | Low risk | Allocation of concealment was performed using computer generated random numbers in sealed opaque envelopes corresponding to either RAMIE or open surgery. |
|  | Blinding of participants and personnel (performance bias) | Low risk | No blinding, but the survival outcome was not likely to be influenced by lack of blinding. |
|  | Blinding of outcome assessment (detection bias) | Low risk | No blinding, but the survival outcome was not likely to be influenced by lack of blinding. |
|  | Incomplete outcome data (attrition bias) | Low risk | Censoring was unlikely to introduce bias for survival outcome. |
|  | Selective reporting (reporting bias) | Low risk | All prespecified endpoint outcomes were reported. |
|  | Other bias | Low risk | All participants in the ITT received surgical resection. The study appeared to be free of other sources of bias. |
| TIME (Straatman, 2017)^24^ | Random sequence generation (selection bias) | Unclear risk | Insufficient information available to permit a judgement of low risk or high risk. Because the method of sequence generation is not described. |
|  | Allocation concealment (selection bias) | Low risk | Central randomization, participants and investigators can’t foresee assignment. |
|  | Blinding of participants and personnel (performance bias) | Low risk | No blinding, but the survival outcome was not likely to be influenced by lack of blinding. |
|  | Blinding of outcome assessment (detection bias) | Low risk | No blinding, but the survival outcome was not likely to be influenced by lack of blinding. |
|  | Incomplete outcome data (attrition bias) | Low risk | Censoring was unlikely to introduce bias for survival outcome. |
|  | Selective reporting (reporting bias) | Low risk | All prespecified endpoint outcomes were reported. |
|  | Other bias | Low risk | 55/59 (93%) participants in the arm MIE and 52/56 (93%) participants in the arm Open Esophagectomy received resection. The study appeared to be free of other sources of bias. |
| Stahl, 2005 (excluded)^27^ | Random sequence generation (selection bias) | Unclear risk | Insufficient information available to permit a judgement of low risk or high risk. Because the method of sequence generation is not described. |
|  | Allocation concealment (selection bias) | Low risk | Central randomization, participants and investigators can’t foresee assignment. |
|  | Blinding of participants and personnel (performance bias) | Low risk | No blinding, but the survival outcome was not likely to be influenced by lack of blinding. |
|  | Blinding of outcome assessment (detection bias) | Low risk | No blinding, but the survival outcome was not likely to be influenced by lack of blinding. |
|  | Incomplete outcome data (attrition bias) | Low risk | Censoring was unlikely to introduce bias for survival outcome. |
|  | Selective reporting (reporting bias) | Low risk | All prespecified endpoint outcomes were reported. |
|  | Other bias | High risk | 57/86 (66%) participants in the arm Induction CT + NCRT (40Gy) + surgery received resection. The low resection rate would lead to high risk of bias in the outcome assessment. |

Abbreviation: NCRT, neoadjuvant chemoradiotherapy; NCT, neoadjuvant chemotherapy; ECX, epirubicin, cisplatin and capecitabine; CF, cisplatin and fluorouracil; ACF, cisplatin, fluorouracil and adriamycin; CT, chemotherapy; ITT, intention-to-treatment population; RAMIE, robot-assisted minimally invasive thoracolaparoscopic esophagectomy.

**References**

1. Yang H, Liu H, Chen Y, Zhu C, Fang W, Yu Z, et al. Neoadjuvant chemoradiotherapy followed by surgery versus surgery alone for locally advanced squamous cell carcinoma of the esophagus (NEOCRTEC5010): a phase III multicenter, randomized, open-label clinical trial. J Clin Oncol. 2018;36(27):2796-2803. https://doi.org/10.1200/JCO.2018.79.1483.
2. Shapiro J, van Lanschot JJB, Hulshof M, van Hagen P, van Berge Henegouwen MI, Wijnhoven BPL, et al. Neoadjuvant chemoradiotherapy plus surgery versus surgery alone for oesophageal or junctional cancer (CROSS): long-term results of a randomised controlled trial. Lancet Oncol. 2015;16(9):1090-1098. https://doi.org/10.1016/S1470-2045(15)00040-6.
3. Mariette C, Dahan L, Mornex F, Maillard E, Thomas PA, Meunier B, et al. Surgery alone versus chemoradiotherapy followed by surgery for stage I and II esophageal cancer: final analysis of randomized controlled phase III trial FFCD 9901. J Clin Oncol. 2014;32(23):2416-2422. https://doi.org/10.1200/JCO.2013.53.6532.
4. Lv J, Cao XF, Zhu B, Ji L, Tao L, Wang DD. Long-term efficacy of perioperative chemoradiotherapy on esophageal squamous cell carcinoma. World J Gastroenterol. 2010;16(13):1649-1654.https://doi.org/10.3748/wjg.v16.i13.1649.
5. Burmeister BH, Smithers BM, Gebski V, Fitzgerald L, Simes RJ, Devitt P, et al. Surgery alone versus chemoradiotherapy followed by surgery for resectable cancer of the oesophagus: a randomised controlled phase III trial. Lancet Oncol. 2005;6(9):659-668. https://doi.org/10.1016/S1470-2045(05)70288-6.
6. Urba SG, Orringer MB, Turrisi A, Iannettoni M, Forastiere A, Strawderman M. Randomized trial of preoperative chemoradiation versus surgery alone in patients with locoregional esophageal carcinoma. J Clin Oncol. 2001;19(2):305-313. https://doi.org/10.1200/JCO.2001.19.2.305.
7. Bosset JF, Gignoux M, Triboulet JP, Tiret E, Mantion G, Elias D, et al. Chemoradiotherapy followed by surgery compared with surgery alone in squamous-cell cancer of the esophagus. N Engl J Med. 1997;337(3):161-167. https://doi.org/10.1056/NEJM199707173370304.
8. Walsh TN, Noonan N, Hollywood D, Kelly A, Keeling N, Hennessy TP. A comparison of multimodal therapy and surgery for esophageal adenocarcinoma. N Engl J Med. 1996;335(7):462-467. https://doi.org/10.1056/NEJM199608153350702.
9. Boonstra JJ, Kok TC, Wijnhoven BP, van Heijl M, van Berge Henegouwen MI, Ten Kate FJ, et al. Chemotherapy followed by surgery versus surgery alone in patients with resectable oesophageal squamous cell carcinoma: long-term results of a randomized controlled trial. BMC Cancer. 2011;11:181. https://doi.org/10.1186/1471-2407-11-181.
10. Ychou M, Boige V, Pignon JP, Conroy T, Bouché O, Lebreton G, et al. Perioperative chemotherapy compared with surgery alone for resectable gastroesophageal adenocarcinoma: an FNCLCC and FFCD multicenter phase III trial. J Clin Oncol. 2011;29(13):1715-1721. https://doi.org/10.1200/JCO.2010.33.0597.
11. Allum WH, Stenning SP, Bancewicz J, Clark PI, Langley RE. Long-term results of a randomized trial of surgery with or without preoperative chemotherapy in esophageal cancer. J Clin Oncol. 2009;27(30):5062-5067. https://doi.org/10.1200/JCO.2009.22.2083.
12. Kelsen DP, Winter KA, Gunderson LL, Mortimer J, Estes NC, Haller DG, et al. Long-term results of RTOG trial 8911 (USA Intergroup 113): a random assignment trial comparison of chemotherapy followed by surgery compared with surgery alone for esophageal cancer. J Clin Oncol. 2007;25(24):3719-3725. https://doi.org/10.1200/JCO.2006.10.4760.
13. Law S, Fok M, Chow S, Chu KM, Wong J. Preoperative chemotherapy versus surgical therapy alone for squamous cell carcinoma of the esophagus: a prospective randomized trial. J Thorac Cardiovasc Surg. 1997;114(2):210-217. https://doi.org/10.1016/S0022-5223(97)70147-8.
14. Ando N, Kato H, Igaki H, Shinoda M, Ozawa S, Shimizu H, et al. A randomized trial comparing postoperative adjuvant chemotherapy with cisplatin and 5-fluorouracil versus preoperative chemotherapy for localized advanced squamous cell carcinoma of the thoracic esophagus (JCOG9907). Ann Surg Oncol. 2012;19(1):68-74. https://doi.org/10.1245/s10434-011-2049-9.
15. Zhao Y, Dai Z, Min W, Sui X, Kang H, Zhang Y, et al. Perioperative versus preoperative chemotherapy with surgery in patients with resectable squamous cell carcinoma of esophagus: a phase III randomized trial. J Thorac Oncol. 2015;10(9):1349-1356. https://doi.org/10.1097/JTO.0000000000000612.
16. Ajani JA, Xiao L, Roth JA, Hofstetter WL, Walsh G, Komaki R, et al. A phase II randomized trial of induction chemotherapy versus no induction chemotherapy followed by preoperative chemoradiation in patients with esophageal cancer. Ann Oncol. 2013;24(11):2844-2849. https://doi.org/10.1093/annonc/mdt339.
17. Stahl M, Walz MK, Riera-Knorrenschild J, Stuschke M, Sandermann A, Bitzer M, et al. Preoperative chemotherapy versus chemoradiotherapy in locally advanced adenocarcinomas of the oesophagogastric junction (POET): long-term results of a controlled randomised trial. Eur J Cancer. 2017;81:183-190. https://doi.org/10.1016/j.ejca.2017.04.027.
18. von Döbeln GA, Klevebro F, Jacobsen AB, Johannessen HO, Nielsen NH, Johnsen G, et al. Neoadjuvant chemotherapy versus neoadjuvant chemoradiotherapy for cancer of the esophagus or gastroesophageal junction: long-term results of a randomized clinical trial. Dis Esophagus. 2019;32(2). https://doi.org/10.1093/dote/doy078.
19. Alderson D, Cunningham D, Nankivell M, Blazeby JM, Griffin SM, Crellin A, et al. Neoadjuvant cisplatin and fluorouracil versus epirubicin, cisplatin, and capecitabine followed by resection in patients with oesophageal adenocarcinoma (UK MRC OE05): an open-label, randomised phase 3 trial. Lancet Oncol. 2017;18(9):1249-1260. https://doi.org/10.1016/S1470-2045(17)30447-3.
20. Yamasaki M, Yasuda T, Yano M, Hirao M, Kobayashi K, Fujitani K, et al. Multicenter randomized phase II study of cisplatin and fluorouracil plus docetaxel (DCF) compared with cisplatin and fluorouracil plus Adriamycin (ACF) as preoperative chemotherapy for resectable esophageal squamous cell carcinoma (OGSG1003). Ann Oncol. 2017;28(1):116-120. https://doi.org/10.1093/annonc/mdw439.
21. Ruhstaller T, Thuss-Patience P, Hayoz S, Schacher S, Knorrenschild JR, Schnider A, et al. Neoadjuvant chemotherapy followed by chemoradiation and surgery with and without cetuximab in patients with resectable esophageal cancer: a randomized, open-label, phase III trial (SAKK 75/08). Ann Oncol. 2018;29(6):1386-1393. https://doi.org/10.1093/annonc/mdy105.
22. Cunningham D, Stenning SP, Smyth EC, Okines AF, Allum WH, Rowley S, et al. Peri-operative chemotherapy with or without bevacizumab in operable oesophagogastric adenocarcinoma (UK Medical Research Council ST03): primary analysis results of a multicentre, open-label, randomised phase 2-3 trial. Lancet Oncol. 2017;18(3):357-370. https://doi.org/10.1016/S1470-2045(17)30043-8.
23. van der Sluis PC, van der Horst S, May AM, Schippers C, Brosens LAA, Joore HCA, et al. Robot-assisted minimally invasive thoracolaparoscopic esophagectomy versus open transthoracic esophagectomy for resectable esophageal cancer: a randomized controlled trial. Ann Surg. 2019;269(4):621-630. https://doi.org/10.1097/SLA.0000000000003031.
24. Straatman J, van der Wielen N, Cuesta MA, Daams F, Roig Garcia J, Bonavina L, et al. Minimally invasive versus open esophageal resection: three-year follow-up of the previously reported randomized controlled trial: the time trial. Ann Surg. 2017;266(2):232-236. <https://doi.org/10.1097/SLA.0000000000002171>.
25. Lee JL, Park SI, Kim SB, Jung HY, Lee GH, Kim JH, et al. A single institutional phase III trial of preoperative chemotherapy with hyperfractionation radiotherapy plus surgery versus surgery alone for resectable esophageal squamous cell carcinoma. Ann Oncol. 2004;15(6):947-954. <https://doi.org/10.1093/annonc/mdh219>.
26. DeWitt JM, Murthy SK, Ardhanari R, DuVall GA, Wallner G, Litka P, et al. EUS-guided paclitaxel injection as an adjunctive therapy to systemic chemotherapy and concurrent external beam radiation before surgery for localized or locoregional esophageal cancer: a multicenter prospective randomized trial. Gastrointest Endosc. 2017;86(1):140-149. https://doi.org/10.1016/j.gie.2016.11.017.
27. Stahl M, Stuschke M, Lehmann N, Meyer HJ, Walz MK, Seeber S, et al. Chemoradiation with and without surgery in patients with locally advanced squamous cell carcinoma of the esophagus. J Clin Oncol. 2005;23(10):2310-2317. https://doi.org/10.1200/JCO.2005.00.034.

**Supplemental Table 3. Subgroup analysis of the correlation between PFS and OS.**

| Subgroup | Trial-level correlation | | Neoadjuvant treatment arm-level correlation | | | | |
| --- | --- | --- | --- | --- | --- | --- | --- |
|  | HR | Δ median survival | Median survival | 1-year PFS | 2-year PFS | 3-year PFS | 5-year PFS |
| ***Neoadjuvant strategy*** | | | | | | | |
| NCRT (n = 14) | 0.64 (0.03-0.98) | 0.80 (0.52-0.97) | 0.96 (0.84-0.99) | 0.82 (0.53-0.96) | 0.88 (0.64-0.98) | 0.88 (0.65-0.97) | 0.93 (0.84-0.98) |
| NCT (n =10) | 0.89 (0.56-0.99) | 0.82 (-1.00-1.00) | 0.86 (0.57-0.99) | 0.76 (0.44-0.99) | 0.96 (0.91-0.99) | 0.95 (0.92-0.99) | 0.97 (0.92-0.99) |
| ***Pathological type*** | | | | | | | |
| SCC (n = 8) | 0.001 (-0.78-0.84) | 0.94 (-0.59-1.00) | 0.99 (0.72-1.00) | 0.73 (0.38-0.97) | 0.89 (0.60-0.99) | 0.90 (0.60-0.99) | 0.93 (0.82-0.99) |
| AC (n = 5) | 0.99 (0.99-1.00) | NA | 0.55 (-1.00-1.00) | NA | NA | NA | NA |
| SCC and AC mixed (n = 11) | 0.91 (0.64-0.99) | 0.85 (-0.31-1.00) | 0.92 (0.83-0.98) | 0.94 (0.83-0.98) | 0.97 (0.93-0.99) | 0.96 (0.93-0.99) | 0.96 (0.89-0.99) |
| ***Publication year*** | | | | | | | |
| 1996-2010 (n = 8) | 0.10 (-1.00-1.00) | 0.43 (-1.00-1.00) | 0.93 (0.25-1.00) | 0.92 (0.63-0.99) | 0.95 (0.69-1.00) | 0.92 (0.57-1.00) | 0.77 (0.64-0.99) |
| 2011-2019 (n = 16) | 0.90 (0.65-0.97) | 0.84 (0.69-0.97) | 0.97(0.78-1.00) | 0.74 (0.40-0.92) | 0.95 (0.88-0.99) | 0.94 (0.87-0.99) | 0.95 (0.90-0.99) |
| ***Overall*** (n = 24) | 0.82 (0.42-0.97) | 0.83 (0.54-0.96) | 0.97 (0.85-0.99) | 0.83 (0.63-0.94) | 0.93 (0.81-0.98) | 0.93 (0.82-0.98) | 0.95 (0.89-0.98) |

Abbreviation: AC, adenocarcinoma; HR, hazard ratio; NA, not available; NCRT, neoadjuvant chemoradiotherapy; NCT, neoadjuvant chemotherapy; PFS, progression-free survival; SCC, squamous cell carcinoma.

**
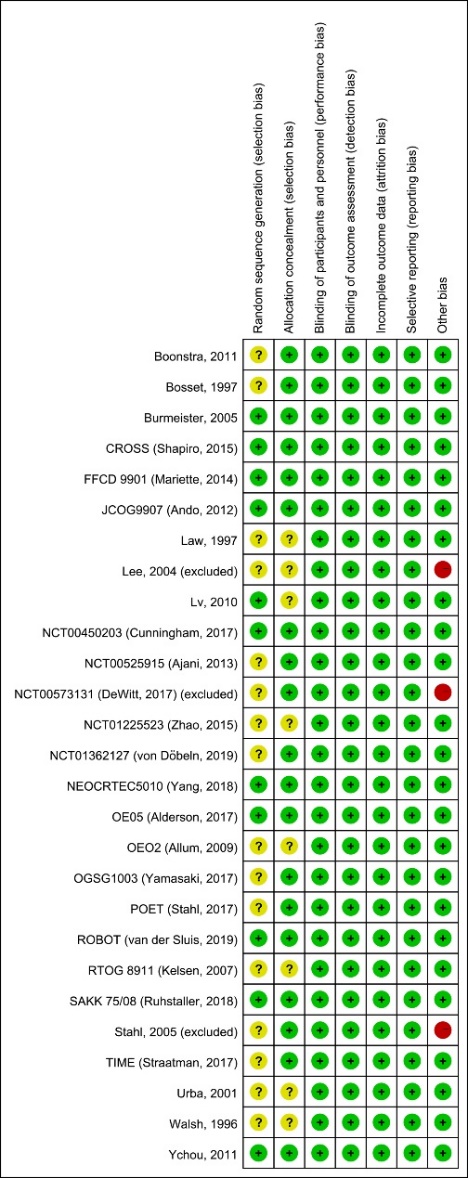
**

**Supplemental Figure 1** Summary of risk of bias in RCTs. “+” (green), “?” (yellow), and “−” (red) represent low, unclear, and high risk of bias, respectively. RCT, randomized controlled trial.


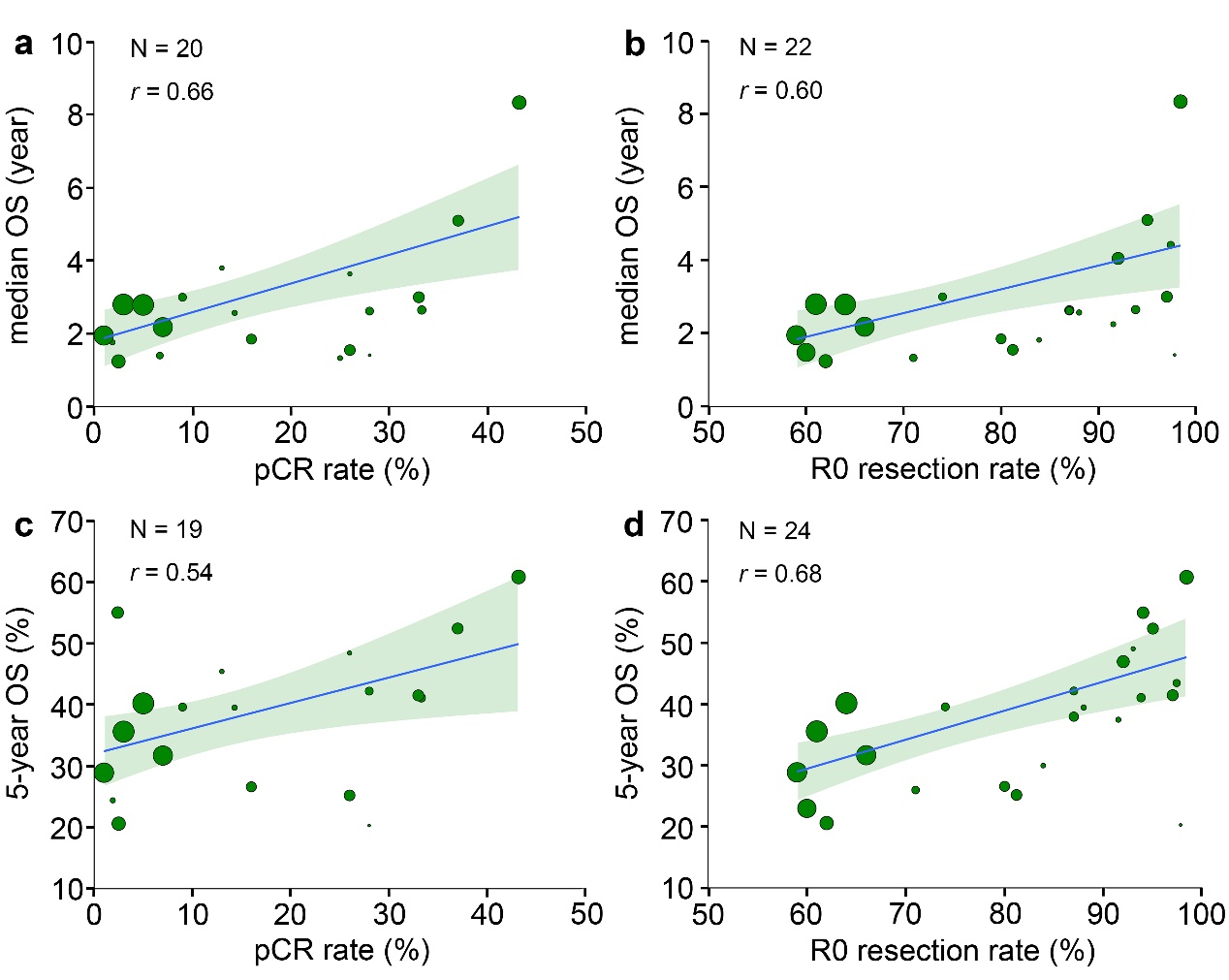


**Supplemental Figure 2** Neoadjuvant treatment arm-level correlation among pCR rate, R0 resection rate and OS. The neoadjuvant treatment arm-level association between **(A)** pCR rate and median OS, **(B)** R0 resection rate and median OS, **(C)** pCR rate and 5-year OS, and **(D)** R0 resection rate and 5-year OS. Circle size was proportional to the number of patients in each treatment arm. The solid blue line indicates the fitted weighted linear regression line, the light green zone represents its 95% CI, and *r* indicates the correlation coefficient. pCR, pathological complete response; OS, overall survival; CI, confidence interval.
